# Premenstrual Disorders in Adolescents: An Interdisciplinary Perspective

**DOI:** 10.3390/jcm15187046

**Published:** 2026-09-11

**Authors:** Krzysztof Dobrzeniecki, Monika Kacprzak, Dobrochna Stachecka, Kornelia Sarnowska, Witold Włodzimierz Kędzia, Małgorzata Mizgier, Magdalena Pisarska-Krawczyk, Katarzyna Plagens-Rotman, Witold Mirosław Kędzia, Justyna Opydo-Szymaczek, Grażyna Jarząbek-Bielecka

**Affiliations:** 1 Department of General and Developmental Sexology at the Gynecology Clinic, Poznan University of Medical Sciences, 61-701 Poznan, Polandkpr.pielegniarstwopolskie@interia.eu (K.P.-R.); witold.kedzia@ump.edu.pl (W.M.K.); grajarz@tlen.pl (G.J.-B.); 2 Department of Sports Dietetics, Faculty of Health Sciences, Poznan University of Physical Education, 61-871 Poznan, Poland; mizgier@awf.poznan.pl; 3 Department of Nursing, The President Stanislaw Wojciechowski Calisia University, 62-800 Kalisz, Poland; magmp@op.pl; 4 Department of Pediatric Dentistry, Poznan University of Medical Sciences, 61-701 Poznan, Poland; jopydo@ump.edu.pl

**Keywords:** premenstrual syndrome, PMS, premenstrual dysphoric disorder, PMDD, premenstrual disorders, adolescents, puberty

## Abstract

**Background:** Premenstrual disorders (PMDs), including premenstrual syndrome and premenstrual dysphoric disorder, are common conditions that may substantially affect adolescents’ physical and psychological well-being. However, their presentation and management during adolescence are complicated by reproductive-axis maturation, overlap with other medical conditions, and limited adolescent-specific evidence. This review aimed to provide an interdisciplinary perspective on PMDs in adolescents, integrating biological, psychological, developmental, clinical, and sociocultural aspects. **Methods:** A structured narrative review was conducted. Publications addressing the neuroendocrine and developmental mechanisms, clinical manifestations, psychosocial consequences, diagnosis, and treatment of PMDs were considered, with adolescent-specific evidence prioritized where available. **Results:** Current evidence suggests that PMDs are associated with altered sensitivity to physiological ovarian steroid fluctuations and their neuroactive effects rather than abnormal circulating hormone concentrations alone. In adolescents, PMDs may present with diverse somatic, affective, cognitive, and behavioral symptoms and are associated with impaired quality of life, school functioning, interpersonal relationships, and psychological well-being. Diagnostic assessment remains challenging because symptoms may overlap with normal pubertal changes and psychiatric disorders. Prospective monitoring of symptom cyclicity and functional impairment is therefore essential. Pharmacological treatments, particularly selective serotonin reuptake inhibitors and selected combined oral contraceptives, represent important therapeutic options, while psychological, lifestyle, physical activity, and physiotherapeutic interventions may provide additional benefits. However, most treatment evidence is derived from adult populations. **Conclusions:** PMDs in adolescents should be conceptualized as multidimensional conditions requiring an interdisciplinary approach integrating gynecological, psychiatric, psychological, endocrinological, primary care, and lifestyle perspectives. Greater recognition and developmentally appropriate assessment may facilitate earlier diagnosis and individualized management. Further prospective, adolescent-specific research is needed to clarify the biological and psychosocial determinants of PMDs and establish evidence-based strategies for their multidisciplinary management.

## 1. Introduction

Premenstrual disorders (PMDs) encompass a spectrum of recurrent physical, psychological, cognitive, and behavioral symptoms that emerge during the late luteal phase of the menstrual cycle and improve or resolve with the onset of menstruation. Among these conditions, premenstrual syndrome (PMS) and premenstrual dysphoric disorder (PMDD) represent distinct but overlapping clinical entities [1]. Beyond their cyclical symptomatology, PMDs can have profound psychological and functional consequences, affecting quality of life, daily functioning, interpersonal experiences, and women’s sense of self [2].

The clinical presentation of PMS encompasses a broad spectrum of symptoms. Psychological manifestations commonly include anxiety, irritability, emotional lability, and depressed mood, whereas somatic symptoms frequently involve headaches, abdominal bloating, mastalgia and fatigue. In severe cases, symptom burden may substantially impair daily functioning, interpersonal relationships, and overall psychosocial well-being [3]. PMDD is a more narrowly defined premenstrual disorder characterized predominantly by affective symptoms and clinically significant functional impairment [4]. It is recognized as a distinct diagnostic entity within the Diagnostic and Statistical Manual of Mental Disorders, Fifth Edition (DSM-5), and is classified among depressive disorders.

Adolescence, defined by the World Health Organization as the period between 10 and 19 years of age, is characterized by substantial biological, psychological, and social development [5]. The reported prevalence of PMDs varies considerably across studies, depending on the population and diagnostic criteria used. Among Korean adolescent girls aged 14–17 years, the prevalence of PMS was 70.5% [6], whereas a multicenter study of adolescents aged 13–21 years reported moderate-to-severe PMS in 14.5% and PMDD in 4.1% [7]. Similarly, among Japanese collegiate athletes, moderate-to-severe PMS and PMDD were identified in 8.6% and 2.9%, respectively [8]. In a population-based Swiss sample of women aged 15–54 years, 10.3% met criteria for PMS and 3.1% for PMDD [9], highlighting the substantial variation in prevalence estimates across populations and assessment approaches.

The consequences of PMDs extend beyond cyclical symptoms and may interfere with adolescents’ daily functioning, educational performance, and psychosocial well-being. Associations with impaired concentration and academic performance, health-risk behaviors, and adverse mental health outcomes underscore the potential clinical significance of PMDs during the developmental period [10,11,12,13,14]. These potential consequences highlight the importance of improving recognition and understanding of PMDs in adolescents, particularly given the developmental and multidisciplinary factors that may influence their presentation and management.

Although women aged 25–34 years represent the group most likely to seek medical attention for PMS symptoms, clinical histories indicate that symptoms frequently precede diagnosis and treatment by several years, in some cases by as much as a decade [3]. This delay between symptom onset and clinical recognition suggests that premenstrual symptoms may emerge during adolescence or early adulthood but remain unrecognized, normalized, or inadequately managed for years. Understanding PMS during adolescence is therefore particularly important for promoting earlier recognition and appropriate management.

Adolescence represents a distinct developmental stage characterized by substantial biological and psychological changes. The maturation of the reproductive system during this period is accompanied by age-specific gynecological conditions and overlapping clinical presentations, which may complicate the recognition and management of reproductive health disorders [15]. As a result, PMS-related complaints may be difficult to distinguish from physiological pubertal changes or emerging psychiatric conditions [16]. This diagnostic complexity may contribute to underrecognition, delayed diagnosis, and inadequate treatment, potentially increasing the long-term burden of the disorder. Despite the clinical significance of PMDs during adolescence, important gaps remain in the evidence base. While recent reviews [17,18,19] have summarized the epidemiology, diagnosis, pathophysiology, and management of PMS and PMDD in adolescents, a comprehensive developmental and interdisciplinary perspective integrating neuroendocrine mechanisms with psychological, psychosocial, interpersonal, and broader health-related factors remains limited. Such an approach is clinically important because the multifaceted presentation of PMDs may require coordinated assessment and management across gynecological, psychiatric, psychological, and primary care settings to facilitate accurate recognition, address comorbidities, and tailor treatment to the individual adolescent.

**Figure 1:**
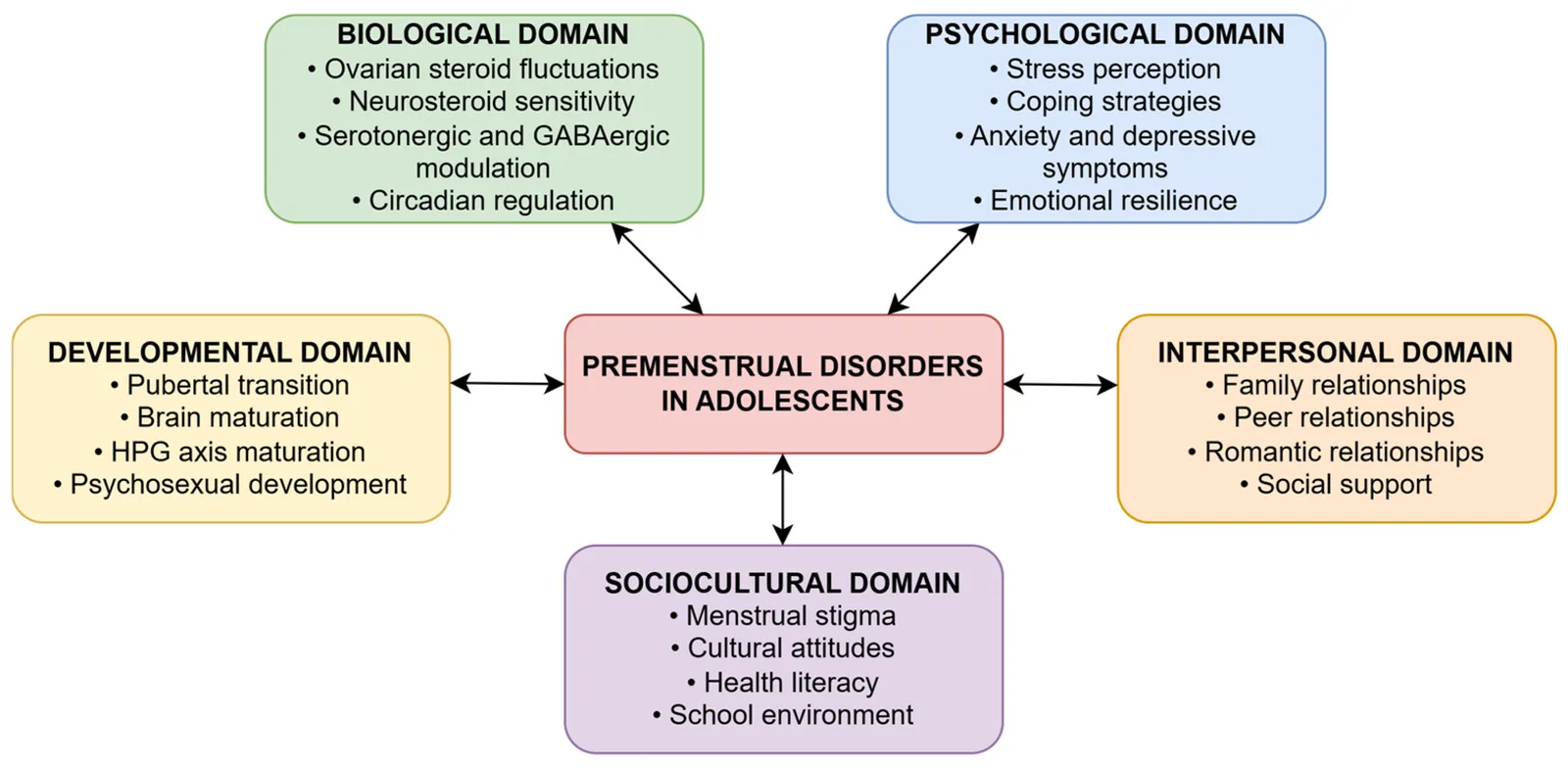
Integrated framework of biological, psychological, interpersonal, sociocultural and developmental factors involved in adolescent PMDs.

The objective of this review is to provide a comprehensive and interdisciplinary overview of PMDs in adolescents. By integrating evidence from gynecology, psychiatry, psychology, endocrinology, and public health, we examine the neuroendocrine and developmental background of PMDs, their clinical manifestations, psychosocial consequences, diagnostic challenges, and current therapeutic approaches. In addition, we highlight key gaps in the existing literature and propose a broader understanding of PMDs in adolescence as multidimensional conditions shaped by interacting biological, psychological, interpersonal, developmental, and sociocultural factors (Figure 1).

## 2. Methods

This review is presented as a narrative review, a format chosen to provide an interdisciplinary perspective on PMDs. Such an approach allows for the integration of evidence from diverse fields and consideration of the biological, developmental, psychological, clinical, and psychosocial aspects of PMDs in adolescence. A narrative approach was adopted because the primary objective was to provide an integrative interpretation of heterogeneous evidence across these domains rather than to address a narrowly defined question through systematic or quantitative synthesis. The review draws on a broad range of scientific literature, including original research studies, systematic reviews, meta-analyses, observational and clinical studies, as well as relevant clinical guidelines and expert position statements. The literature was considered relevant when it addressed the presentation, determinants, pathophysiology, assessment, diagnosis, or management of PMDs, with particular emphasis on adolescent populations. Conference abstracts, case reports, non-peer-reviewed publications, and articles considered outside the scope of the review were excluded. Publications in English or Polish were considered, reflecting the linguistic capabilities of the research team.

Adolescent-specific evidence was prioritized whenever available. However, studies involving broader reproductive-age or adult populations were also considered when adolescent-specific evidence was limited or unavailable, or when such studies provided relevant contextual, comparative, or foundational information for understanding PMDs in adolescents. Evidence derived from adult populations was used primarily to provide biological, clinical, or contextual background and was not assumed to be directly generalizable to adolescents.

The literature search was conducted using the PubMed, Embase, Scopus, and Web of Science databases between May and June 2026. The search strategy combined keywords and their relevant variations related to PMDs and adolescence, including premenstrual syndrome, PMS, premenstrual dysphoric disorder, PMDD, premenstrual disorders, adolescents, adolescence, and the hypothalamic-pituitary-gonadal axis. Search terms and strategies were adapted to the specific subject matter addressed in individual sections of the review, consistent with the narrative nature of the synthesis. The final database search was conducted on 26 June 2026. Publications from the preceding 50 years were considered for inclusion.

## 3. Neuroendocrine and Developmental Background

The pathophysiology of PMS remains incompletely understood, with several biological mechanisms proposed to contribute to the development of symptoms. Classical models have linked PMS to cyclical fluctuations in ovarian sex steroids, particularly estrogen and progesterone, with mood deterioration and anxiety associated with changes in their concentrations during the menstrual cycle. More recent evidence has shifted attention toward individual sensitivity and adaptive responses to hormonal fluctuations, including the effects of progesterone metabolites such as allopregnanolone on GABAergic neurotransmission [20]. Moreover, PMS symptoms are closely linked to postovulatory hormonal events and ovarian activity. Evidence from ovulatory and anovulatory cycles indicates that symptoms may disappear in the absence of ovulation, supporting a role for postovulatory ovarian activity, particularly corpus luteum-derived progesterone, in symptom development [21,22].

However, evidence has challenged the concept that PMS results from abnormal circulating concentrations of ovarian hormones. Bäckström et al. found no differences in serum concentrations of progesterone, estradiol, testosterone, or androstenedione between women with high and low degrees of cyclical mood change. Although negative mood changes consistently developed after ovulation and increased during the luteal phase, their severity was not associated with differences in circulating concentrations of these hormones [23]. Similarly, luteal-phase plasma levels of progesterone, allopregnanolone, and pregnanolone did not differ between women with PMS and controls and were not associated with symptom severity [24]. These findings have contributed to the hypothesis that individual sensitivity to physiological hormonal fluctuations, rather than abnormal hormone concentrations alone, may play an important role in the development of PMDs [25].

Support for this concept was provided by studies demonstrating that physiological doses of estradiol and progesterone re-induced symptoms in women with PMS following gonadotropin-releasing hormone (GnRH) agonist-induced ovarian suppression, whereas asymptomatic controls remained unaffected [26]. Similarly, in women with PMDD, symptom recurrence during the initial phase of estradiol and progesterone add-back therapy, followed by remission despite continued exposure to stable hormone levels, suggests that symptom onset may be driven by dynamic changes in ovarian steroids rather than sustained exposure to elevated hormone levels [27]. Consequently, PMS and PMDD are increasingly conceptualized as disorders of altered neurobiological sensitivity to normal cyclical ovarian steroid changes rather than disorders of hormone excess or deficiency.

Beyond circulating ovarian steroid concentrations, attention has also been directed toward the neuroactive metabolites of progesterone. Although PMS and PMDD symptoms are not associated with defined concentrations of progesterone or other gonadal hormones, progesterone metabolites produced by the corpus luteum and in the brain may influence central nervous system function through their effects on GABAergic neurotransmission [22]. Particular interest has centered on allopregnanolone, a potent neuroactive metabolite of progesterone that acts as a positive allosteric modulator of γ-aminobutyric acid type A receptors [20]. Neuroactive steroids can modulate neurotransmitter receptors through genomic and non-genomic mechanisms and may influence stress responses and a broad range of behavioral functions [28].

Another proposed mechanism involves alterations in serotonergic neurotransmission. Serotonergic dysfunction has been implicated in symptoms commonly observed in PMS, including depressed mood, anxiety, irritability, impaired impulse control, and difficulties with concentration, although serotonergic mechanisms are unlikely to fully account for the complex symptomatology of PMS [29]. In women with PMS, lower whole-blood serotonin concentrations have been reported during the late luteal phase compared with earlier phases of the menstrual cycle, supporting a possible role for altered serotonin metabolism in symptom development [30]. Experimental studies further suggest that ovarian steroids can modulate serotonergic signaling; for example, estradiol has been shown to alter serotonin receptor density in specific brain regions in female rats [31]. More recent evidence suggests that cyclical fluctuations in gonadal hormones may influence serotonergic neurotransmission, providing a potential link between ovarian hormonal changes and the affective and behavioral symptoms of PMDs [32]. 

Additional support for serotonergic involvement comes from the well-established efficacy of selective serotonin reuptake inhibitors (SSRIs) and serotonin-norepinephrine reuptake inhibitors in PMS and PMDD treatment [33]. Interestingly, symptom improvement often occurs more rapidly than would be expected based on traditional models of antidepressant action. This observation suggests that SSRIs may exert therapeutic effects through mechanisms extending beyond conventional enhancement of serotonergic transmission, potentially involving interactions with neurosteroid synthesis and GABAergic signaling [20,34].

Sleep and circadian disturbances may represent an important component of PMS symptomatology. Women with PMS commonly report poor sleep, insomnia, and daytime sleepiness during the luteal phase. In a recent study of female nursing students, insomnia was associated with almost fourfold higher odds of PMS symptoms (OR = 3.93, 95% CI 1.14–13.59) [35]. Circadian alterations, including delayed sleep–wake rhythms and changes in melatonin secretion, have also been reported in PMS/PMDD, although their precise role remains uncertain [36]. This relationship may be particularly relevant during adolescence, a developmental period characterized by a physiologically driven delay in circadian timing, later sleep schedules, and substantial differences between weekday and weekend sleep patterns [37]. In a cross-sectional study of Korean high school girls conducted during the COVID-19 lockdown, poor sleep quality and short sleep duration were associated with PMS, while associations with late bedtime and wake-up time were less robust after adjustment for covariates; among the sleep characteristics examined, sleep quality emerged as the most important risk factor for PMS [38].

Sleep disturbances are frequently reported in women with PMS and may be closely linked to the circadian disruption observed in the disorder [39,40]. Although current evidence supports an association between sleep disruption and PMS, the precise role of circadian dysregulation in adolescent populations remains insufficiently understood. Available studies consistently indicate an association between PMS and several dimensions of sleep disturbance, including poor sleep quality, insomnia symptoms, daytime sleepiness, and shorter sleep duration, suggesting that sleep disturbances may be closely linked to the clinical expression of PMS [35,41,42]. In addition, emerging evidence in PMDD indicates that sleep may vary across the menstrual cycle and may represent a potentially relevant physiological correlate of affective symptom fluctuations [43]. However, objective measures of sleep timing and circadian function remain limited, and the directionality of the relationship between sleep disturbances and PMDs requires further investigation. 

Kiss et al. demonstrated an association between sleep disturbances and menstrual problems in early adolescent girls, reporting that shorter sleep duration and greater sleep disturbance were associated with more severe premenstrual symptoms [44]. Similarly, a recent study among adolescents aged 12–18 years found that poorer sleep quality was significantly associated with greater PMS severity, suggesting a potential relationship between PMS and sleep disturbances in this population [45]. These findings indicate that sleep characteristics are associated with the severity of menstrual problems, including premenstrual symptoms, from early adolescence, highlighting sleep as a potentially relevant and modifiable factor in the assessment of adolescent PMS [44].

Irregular menstrual cycles related to anovulation are well recognized during the first few years following menarche; however, the normal developmental trajectory through which the reproductive axis progresses from predominantly anovulatory to mature ovulatory cycles during adolescence remains poorly characterized. In particular, the final stages of reproductive axis maturation and the transition toward established ovulatory cyclicity remain insufficiently understood, despite their potential importance for understanding reproductive health across the lifespan. This knowledge gap is particularly relevant to PMDs, which are closely linked to cyclical ovarian activity, as the establishment and maturation of ovulatory cycles may represent an important developmental context for the emergence and expression of premenstrual symptoms [16,46,47]. Further research into the maturation of the reproductive axis during adolescence may therefore help clarify how physiological reproductive development relates to the onset and clinical manifestation of PMDs.

These developmental considerations raise important questions regarding the applicability of adult PMDs models to adolescent populations. It remains unclear whether mechanisms involving luteal-phase neurosteroid sensitivity, serotonergic modulation, and ovarian steroid fluctuations operate similarly before full maturation of the reproductive axis. Future research should therefore focus on clarifying whether adolescent PMDs follow the same neuroendocrine and ovulatory patterns described in adults and on identifying developmental factors that may contribute to age-specific symptom expression. Addressing these gaps will be essential for improving the understanding, diagnosis, and management of PMDs during adolescence.

## 4. Clinical Manifestations of PMDs

PMDs are characterized by recurrent symptoms that are temporally linked to the menstrual cycle, typically emerging during the premenstrual or luteal phase and improving or remitting after menstruation. Their clinical presentation is heterogeneous, encompassing a broad spectrum of physical, emotional, and behavioral symptoms, with severity ranging from relatively mild, non-distressing symptoms to clinically significant impairment in daily functioning [1,48]. PMDD represents the more severe end of the premenstrual disorder spectrum and is characterized predominantly by dysphoric symptoms and substantial functional impairment [1].

The most common physical symptoms of PMS include abdominal bloating and fatigue, followed by breast tenderness, headache, dizziness, and flushing. Other somatic manifestations may include weight gain, swelling or edema of the extremities, abdominal pain or cramps, and generalized aches and pains. The broad range of physical symptoms contributes to the heterogeneous clinical presentation of PMDs and may be accompanied by psychological and behavioral symptoms [1,49,50]. Fluid retention has historically been proposed as a contributor to premenstrual symptomatology and may underlie subjective sensations of swelling, bloating, and edema during the luteal phase [48,51]. However, objective measurements do not necessarily demonstrate corresponding changes in body weight; Tacani et al. demonstrated significant increases in regional circumference and limb volume during the premenstrual phase without significant changes in body mass or BMI [51]. 

Psychological and behavioral symptoms constitute an important component of PMDs and may substantially affect daily functioning and quality of life. The severity of symptoms varies considerably, and clinically significant PMS or PMDD is distinguished not simply by the presence of individual premenstrual symptoms but by their intensity, cyclical pattern, and impact on functioning. In adolescents, premenstrual symptoms may additionally interfere with school functioning and social interactions, while more severe PMDs have been associated with poorer physical, emotional, and social quality of life [20,52]. Among emotional symptoms, irritability was reported most frequently by adolescents [53]. In the POLKA 18 study of 1545 Polish adolescents, PMS was significantly associated with higher reported stress levels compared with adolescents without PMS [12]. 

In a study by Derman et al., premenstrual symptoms in adolescents were classified into four groups: negative affect, water retention, food-related symptoms, and pain. Negative affect was the most frequently reported group, with stress and nervousness being the predominant manifestations (both 87.6%). Water-retention symptoms were also common, particularly abdominal bloating (70.5%) and fatigue (69.5%), which frequently occurred together. Food-related symptoms included increased appetite and food cravings, each reported by 51.4% of participants. Pain-related symptoms comprised abdominal cramps and headache, reported by 70.5% and 24.8% of adolescents, respectively. Notably, pain was the only symptom group reported more frequently during the menstrual phase than during the luteal phase [54].

Among adolescents, PMDs have been associated with poorer health-related quality of life, with particularly pronounced impairments in emotional and physical role functioning, social functioning, and bodily pain. These findings suggest that PMDs may affect multiple domains of adolescents’ daily functioning and overall well-being, extending beyond the physical symptoms of the menstrual cycle. The impact appears to be greater among adolescents with more severe premenstrual disorders, particularly PMDD, highlighting the importance of recognizing the broader functional and psychosocial burden of PMDs in this population [52]. 

Importantly, the recognition of PMDs in adolescents presents unique challenges. Premenstrual symptoms may be underrecognized during adolescence because many manifestations overlap with normative developmental experiences. The broad emotional, cognitive, and behavioral manifestations of PMS in adolescents, together with its potential impact on social and academic functioning, may make recognition of the cyclical nature of symptoms challenging. This may be compounded by limited awareness of PMS among adolescents, highlighting the importance of careful assessment of menstrual patterns and psychosocial functioning [55]. An additional challenge in adolescents may arise from the considerable variability in PMS symptom severity. Studies in adolescent populations indicate that symptoms may range from mild to severe, with affective manifestations such as irritability, anxiety, and mood changes being particularly common. The presence of relatively mild and nonspecific symptoms may make their clinical significance more difficult to recognize, particularly when they overlap with other emotional and behavioral changes occurring during adolescence [56,57]. Consequently, differentiating pathological premenstrual symptoms from normal pubertal changes remains a significant clinical challenge and may contribute to delayed diagnosis and under-treatment in adolescent populations.

## 5. Psychosocial Aspects of PMDs

### 5.1. Psychological and Social Impact

PMS is increasingly recognized as a condition associated with significant psychological and social consequences. Although PMS is characterized by cyclical physical and emotional symptoms, its impact extends beyond menstrual health and may affect multiple aspects of daily functioning and overall well-being [32]. A study conducted among girls aged 12–18 in the United Arab Emirates reported that menstrual pain was the second main reason for missing school. The study found that PMS had, to some extent, an impact on lifestyle, social interactions, and particularly academic performance [58].

PMS is associated with a broad range of psychological and emotional symptoms, including mood swings, nervousness, irritability, tearfulness, anxiety, and impaired concentration. In Polish adolescents, PMS was significantly associated with higher levels of perceived stress, anxiety, and panic attacks, as well as with suicidal ideation and self-harm [12]. The potential relationship between PMDs and suicidal thoughts and behaviors is further supported by evidence from individuals with PMDD diagnosed prospectively using daily symptom ratings. In a global survey of 599 individuals with such a diagnosis, high lifetime rates of suicidal ideation, suicide attempts, and non-suicidal self-injury were reported, highlighting the importance of systematic assessment of suicide risk in individuals with PMDD [14].

Behavioral factors may also be relevant to the psychosocial burden of PMS. Cigarette smoking has been associated with an increased risk of recurrent PMS, and smoking has been proposed as a potential coping behavior in response to premenstrual dysphoria. Current and former smokers have also reported more pronounced negative menstrual symptoms than nonsmokers, although the direction and nature of this association remain unclear [12,59,60].

The psychological and functional burden of PMDs is also reflected in their impact on health-related quality of life. Among adolescents with PMDD, significantly poorer quality-of-life scores were observed, with particularly pronounced impairments in emotional and physical role functioning, social functioning, and bodily pain. Premenstrual symptoms may also negatively affect psychological health, social interactions, school functioning, and daily life [52].

Overall, the available evidence suggests that PMS and PMDD may significantly affect psychological well-being, social functioning, and quality of life. Their impact extends beyond the physical manifestations of the disorder, highlighting the importance of recognizing PMDs as conditions with meaningful psychosocial consequences.

### 5.2. Sociocultural Perception of Menstruation and PMDs

Although menstruation is a universal biological process, the perception and experience of premenstrual symptoms may be influenced by social, cultural, and ethnic factors [61]. In a survey of adolescent girls in Tanzania, religion-based menstrual restrictions were reported by 52% of Christian girls and 76% of Muslim girls. Restrictions such as prohibitions against praying during menstruation were more commonly reported among Muslim girls [62]. Research among adolescent girls aged 13–19 years in Kerala found that negative attitudes toward menstruation were common and were more frequently reported among girls from Muslim families. Muslim adolescents were also more likely to report severe PMS [63].

Menstrual stigma remains an important global issue that may negatively affect the psychological, social, and educational well-being of women and girls. Evidence from qualitative studies across diverse cultural settings indicates that menstruation is frequently surrounded by stigma, silence, and negative attitudes, which can limit open discussion, access to accurate information, and opportunities to seek support. These sociocultural expectations may contribute to shame, embarrassment, fear of menstrual status being revealed, and distress, while also reinforcing expectations that menstruation should be concealed and that girls should modify their behaviour during menstruation. The effects of stigma may extend beyond perceptions of menstruation itself, influencing confidence, social participation, and psychological well-being. Importantly, the manifestation of menstrual stigma varies across cultural contexts, with restrictions and behavioural expectations differing according to factors such as religion, region, family expectations, and local social norms. In some settings, embarrassment and a lack of supportive healthcare interactions may also discourage women and girls from seeking professional help for menstrual-related problems [64]. Many adolescent girls had limited knowledge of the physiology and mechanism of menstruation, as they had not received detailed information before menarche. Consequently, many described the onset of menarche as shocking and emotionally impactful. Moreover, girls from rural areas were more likely than their urban counterparts to feel embarrassed discussing menstruation-related problems with their mothers or consulting physicians [65]. These findings highlight the need for appropriate menstrual health education to improve adolescents’ knowledge and support effective coping with menstrual-related symptoms, including PMS.

Menstrual stigma may manifest differently across cultural contexts. In the United States, menstrual products are marketed to conceal menstruation from public view, reinforcing expectations that menstrual status should remain invisible. In Nepal, menstruation may be more visible through cultural and religious practices, including abstaining from worship, eating or sleeping separately, or using separate paths. Such practices may be perceived as stigmatizing when they mark menstruating individuals as “impure” [66].

Although the sociocultural influences surrounding menstruation have been relatively well documented, particularly with regard to stigma, restrictive norms, and culturally shaped perceptions, their specific role in PMDs remains insufficiently understood. Further research is therefore needed to clarify how sociocultural context influences the recognition, interpretation, reporting, and management of PMDs, particularly during adolescence and early adulthood.

### 5.3. Interpersonal and Educational Impact

PMDs may substantially affect interpersonal and social functioning during adolescence. In a study of 602 adolescent girls, those with PMDD reported significantly poorer health-related quality of life, with particularly pronounced impairments in social functioning and emotional and physical role functioning. PMDs have also been associated with difficulties in school functioning and social interactions, suggesting that their impact extends beyond physical symptoms to broader psychosocial functioning. Emotional and behavioral symptoms such as irritability and mood swings may further contribute to difficulties in interpersonal relationships and social participation [52].

Similar findings have been reported among adolescents attending child and adolescent psychiatry clinics. In this clinical population, adolescents with PMS had significantly lower health-related quality-of-life scores than those without PMS, with HRQoL progressively deteriorating as PMS severity increased. A high prevalence of coexisting psychiatric disorders was also observed, with anxiety disorders and major depressive disorder being the most common diagnoses. Irritability and anger were among the most frequently reported premenstrual symptoms, further highlighting the relationship between premenstrual symptom severity, emotional well-being, and overall functioning [67]. However, these findings should be interpreted with caution, as they were derived from a clinical psychiatric population and may not be representative of adolescents in the general population. The potential overlap between psychiatric symptoms and premenstrual symptomatology should also be considered when interpreting these findings.

Educational functioning may also be adversely affected by PMDs. Among Thai high school students, PMS was associated with significantly more frequent difficulties with concentration and motivation, poorer individual and collaborative work performance, and lower academic scores [11]. In agreement with these findings, a study conducted among adolescent schoolgirls in the United Arab Emirates found that PMS had a significant negative impact on quality of life, particularly on school performance, with difficulty performing school functions among the most adversely affected domains [58].

The available evidence indicates that PMDs may influence not only physical and emotional well-being but also interpersonal relationships and educational functioning. These consequences may be particularly relevant during adolescence, a developmental period in which academic achievement and peer relationships play a central role in psychosocial development.

### 5.4. Sexual Function: Adult Evidence and the Adolescent Knowledge Gap

Available evidence suggests a potential relationship between PMDs and female sexual functioning, with plausible biological, psychological, and social links between PMDs and female sexual dysfunction. However, the literature remains limited, particularly regarding PMDD, and the available studies are characterized by important methodological limitations. Further research is therefore needed to clarify the relationship between premenstrual symptoms and sexual functioning [68].

Evidence regarding the relationship between PMDs and female sexual functioning remains limited and inconsistent. Conzatti et al. found no significant differences in female sexual function between women with and without PMS in either the luteal or follicular phase, although poorer FSFI scores were associated with the luteal phase [69]. In contrast, a prospective study found that women with PMS were significantly more likely to report sexual concerns, sexuality-related personal distress, and overall sexual difficulties compared with women without PMS [70]. Similarly, a large population-based study found that women with PMS reported lower sexual satisfaction and greater sexual distress than women without PMS, with the presence of PMS remaining negatively associated with sexual satisfaction after adjustment for potential confounders [71].

Evidence specifically examining sexual function in PMDD remains scarce. A narrative review identified only a small number of studies addressing sexual function in women with PMDD and highlighted significant methodological limitations in the available evidence [68]. A randomized controlled trial examining sexual drive and desire in women with PMDD found a trend toward greater improvement in luteal-phase sexual drive and desire with fluoxetine compared with placebo, although the difference did not reach statistical significance [72]. Overall, the available evidence suggests that PMS may be associated with sexual difficulties, lower sexual satisfaction, and greater sexual distress, although findings are inconsistent and evidence specific to PMDD remains limited.

Importantly, these studies involved adult women, and data on sexual functioning in adolescents with PMDs are lacking. This represents an important gap in the literature and highlights the need for prospective studies specifically examining sexual functioning in adolescents with PMDs (Table 1). 

## 6. Diagnostic Challenges

The diagnosis of PMS remains challenging because no objective laboratory test, imaging modality, or biological marker is currently available. Consequently, diagnosis relies primarily on clinical assessment, prospective symptom monitoring, and the exclusion of alternative medical and psychiatric conditions [25,77].

The diagnostic criteria proposed by the American College of Obstetricians and Gynecologists require several conditions to be fulfilled. Symptoms should occur during the premenstrual period, recur in a predictable pattern, and be associated with clinically significant functional impairment. Diagnosis should be supported by prospective daily symptom recording over at least two symptomatic cycles, and other conditions that may account for the symptoms should be excluded [78].

The inclusion of PMDD as an official diagnosis in the DSM-5 represented an important milestone in the classification of PMDs [79]. According to DSM-5 criteria, PMDD is characterized by the presence of at least five symptoms occurring during most menstrual cycles, with characteristic premenstrual symptomatology and clinically significant distress or interference with daily-life activities. Prospective daily symptom ratings over at least two cycles are required for confirmation, while symptoms should not be better explained by another psychiatric disorder, medical condition, or drug effect [80,81]. 

Despite these advances, several controversies remain. One of the most debated aspects of the DSM-5 criteria is the requirement for at least five symptoms. Some authors have argued that this threshold may exclude women who experience substantial symptom-related burden despite having fewer symptoms, whereas lowering the threshold has raised concerns about potentially pathologizing normal physiological menstrual experiences [1,79].

Prospective symptom assessment is an important component of PMDD diagnosis, with DSM-5 criteria requiring daily symptom ratings over at least two cycles. Several instruments have been developed to prospectively assess premenstrual symptoms, including the Daily Record of Severity of Problems, while other validated tools are available for assessing PMDs. Prospective symptom tracking facilitates evaluation of the temporal and cyclical pattern of symptoms, assessment of functional impairment, and differentiation of PMDs from premenstrual exacerbation of other psychiatric disorders. It may also support longitudinal monitoring of symptom changes and treatment response [80,81,82]. Specialized screening tools have also been developed specifically for adolescents, including the Premenstrual Symptoms Screening Tool-Modified for Adolescents (PSST-A). Compared with the original PSST, two items were modified: “work efficiency and productivity” was changed to “school/work efficiency and productivity” and “relationship with coworkers” was changed to “relationship with classmates/friends/coworkers”. The use of tools tailored specifically to adolescents may facilitate more accurate screening and clinical assessment of PMDs in this population [53]. More recent research has further evaluated the psychometric properties of the PSST in adolescent populations. In a study of 939 adolescent girls in Bangladesh, the Bangla version of the Premenstrual Symptoms Screening Tool (PSST) demonstrated excellent internal consistency and satisfactory convergent validity, supporting its use as a screening tool for PMS in this population [83]. However, screening instruments such as the PSST or PSST-A are intended to facilitate screening rather than replace prospective assessment of symptom cyclicity when establishing a diagnosis. 

An important component of the diagnostic process is the exclusion of alternative medical conditions that may mimic the symptoms of PMDs. Clinical evaluation, including gynecological assessment when appropriate, should consider disorders such as thyroid disease, anemia, and endometriosis [1,48,50,84].

Differential diagnosis is further complicated by the substantial overlap between PMD symptoms and psychiatric disorders. Depressive disorders, anxiety disorders, substance use disorders, and alcohol-related problems may present with symptoms similar to those observed in PMS [85]. Moreover, several psychiatric conditions may exhibit premenstrual exacerbation, making it difficult to determine whether symptoms represent a primary premenstrual disorder or cyclical worsening of an existing psychiatric condition [82,84].

Although the available evidence has primarily been derived from adult populations, emerging findings suggest that a similar association may also exist among adolescents with PMS, potentially placing them at an increased risk of mental health problems. However, a research gap remains in this area and further research is needed to clarify and confirm this relationship [12,86].

Another challenge arises from the high prevalence of premenstrual symptoms in the adolescent population. In the study conducted by Vichnin et al., 54% of adolescents reported having PMS but did not meet the predefined symptom and impairment criteria, whereas 31% met the criteria for clinically significant PMS [73]. This finding highlights the importance of standardized diagnostic criteria and prospective assessment in adolescents to distinguish common premenstrual symptoms from clinically significant PMDs and to accurately assess symptom severity, timing, and functional impairment. 

These difficulties may be particularly relevant during adolescence. Although PMS appears to be common in this population, diagnostic assessment remains challenging, as many adolescents report premenstrual symptoms without meeting predefined criteria for clinically significant PMS [73]. In addition, the first years following menarche are frequently characterized by irregular and anovulatory menstrual cycles related to the ongoing maturation of the hypothalamic–pituitary–gonadal axis [16,47]. The normal developmental transition from predominantly anovulatory to mature ovulatory cycles during adolescence remains incompletely understood, representing an additional area of uncertainty in understanding reproductive-axis maturation [47]. Moreover, PMS has historically been underrecognized among adolescents, who may have limited awareness of the condition and its potential impact [55]. These considerations underscore the importance of standardized diagnostic approaches, prospective symptom assessment, and age-appropriate assessment tools for the identification of clinically significant PMDs in adolescents [73,84]. 

Given these challenges, further development of diagnostic approaches remains warranted. Standardized prospective symptom assessment using validated daily symptom diaries remains a key component of diagnosis and may improve diagnostic accuracy; facilitate differentiation between PMS, PMDD, and premenstrual exacerbation of other disorders; and enable systematic monitoring of treatment response over time. Future research should focus on improving diagnostic strategies for adolescents, including further validation of age-appropriate assessment tools and identification of factors that may facilitate earlier recognition of clinically significant PMDs. 

## 7. Therapeutic Approaches

### 7.1. Evidence-Based Pharmacological Treatment

Current pharmacological approaches to PMS and PMDD are largely based on the prevailing neurobiological models of these disorders. Available pharmacological treatments target either cyclical ovarian hormone activity or central serotonergic neurotransmission. The number of studies on the treatment of PMDs in adolescents is currently limited; therefore, current guidelines are based primarily on research conducted in adult populations. SSRIs are considered among the most effective pharmacological treatments for PMDs. A recent systematic review including 34 randomized controlled trials demonstrated that SSRIs significantly reduced overall premenstrual symptom severity compared with placebo. Despite their demonstrated efficacy, SSRIs may be associated with adverse effects, most commonly fatigue, nausea, and somnolence [87]. Studies evaluating SSRIs for PMDs have been conducted primarily in adults, although SSRIs are also used in adolescents. Given the potential risk of suicidal ideation, adolescents and their families should be informed about the black-box warning and the possibility of early activating effects before mood improvement. Careful monitoring for emerging or worsening suicidal ideation or behavior is therefore particularly important during the initial stages of treatment [19].

Another widely used pharmacological strategy involves suppression of ovulation and the associated cyclical fluctuations in ovarian hormones through combined oral contraceptives (COCs). Among hormonal interventions, monophasic COCs have the strongest evidence for the treatment of PMDD, with drospirenone-containing formulations being the most extensively studied. The formulation containing 3 mg of drospirenone and 20 μg of ethinyl estradiol, administered as 24 active pills followed by 4 inactive pills, is currently the only oral contraceptive specifically FDA-approved for PMDD in postmenarchal individuals of any age. Evidence indicates that COCs can improve overall premenstrual symptom severity and functional impairment, while drospirenone-containing formulations may also improve social and interpersonal functioning [19,88]. However, studies in adults have demonstrated substantial placebo responses, highlighting the complexity of evaluating treatment efficacy in premenstrual disorders. Compared with placebo, drospirenone-containing contraceptives have also been associated with a higher frequency of adverse effects, particularly breast pain, nausea, and intermenstrual bleeding [76]. COCs may be particularly beneficial for adolescents who require contraception or who seek management of other menstrual disorders such as heavy menstrual bleeding or dysmenorrhea [19,88].

GnRH agonists represent another therapeutic option. By inducing reversible ovarian suppression, these agents eliminate cyclical ovarian hormone fluctuations and have been shown to improve overall symptom severity and functional impairment [32,50,89]. Although GnRH agonists are used to treat adults, they are not recommended for the treatment of adolescents due to the lack of studies on this topic and concerns regarding potential long-term adverse effects on bone health. Similarly, although they are widely used in adults for the treatment of endometriosis and chronic pelvic pain, they are not recommended for empiric use in adolescents aged 18 years and younger because of concerns about bone health, as they may decrease bone mineral density [19,88,90].

Overall, current evidence supports SSRIs and hormonal therapies, particularly COCs, as the main pharmacological treatments for PMDs. However, mentioned studies were conducted primarily in adult populations. Therefore, further research involving adolescents is needed. Treatment selection should be individualized according to symptom severity, adverse-effect profiles, patient preferences, and the presence of comorbid conditions.

### 7.2. Psychological and Behavioral Therapies

In addition to pharmacological treatment, psychological and behavioral interventions have been increasingly investigated as therapeutic options for PMDs. Among these approaches, cognitive-behavioral therapy (CBT) has received the greatest research attention because of its potential to address emotional symptoms, maladaptive coping strategies, and the functional impairment associated with PMDs [91].

The available evidence regarding the effectiveness of psychological interventions remains heterogeneous. A meta-analysis including 22 studies comparing cognitive-behavioral interventions with serotonergic antidepressants found that both approaches were associated with only modest improvements in symptom severity [92]. In contrast, a more recent systematic review including 32 studies reported significant reductions in PMS symptoms following nonpharmacological interventions, particularly CBT, with some studies describing complete symptom remission. The authors further noted an apparent absence of significant adverse effects and suggested that treatment benefits may be sustained over time [91].

Psychotherapeutic interventions have also been evaluated with respect to emotional symptoms commonly associated with PMS. A meta-analysis of 7 studies demonstrated significant reductions in symptoms of depression and anxiety among women receiving psychotherapy compared with control groups, suggesting that psychological interventions may help reduce the emotional burden associated with PMS [93].

Current evidence suggests that psychological and behavioral interventions, particularly CBT, may represent a valuable component of PMS management. Nevertheless, the studies were conducted primarily in adults; therefore, further research involving adolescents is needed to provide evidence regarding the efficacy of CBT in this population.

### 7.3. Lifestyle and Nutrition

Lifestyle interventions constitute an important component of management of PMDs and are frequently recommended as part of a multimodal treatment strategy. Current evidence suggests that modifiable factors, including regular physical activity, stress management, and dietary patterns, may contribute to the reduction or management of PMD symptoms and improvement in overall well-being. However, the evidence supporting dietary interventions remains limited and heterogeneous [94,95]. Studies conducted in university student populations suggest that lifestyle factors, including dietary habits, physical activity, and tobacco use, are associated with the occurrence and severity of PMS. Furthermore, students with PMS have been reported to have lower overall health-promoting lifestyle scores, with greater symptom severity associated with less favorable lifestyle profiles [75].

Among lifestyle-based interventions, physical activity has received considerable attention. Regular physical activity, including aerobic exercise, yoga, and Pilates, has been associated with reductions in PMS symptom burden and may improve both physical and psychological symptoms. However, the optimal type and dosage of exercise remain uncertain, and further research is needed to establish personalized exercise recommendations [94]. In a cross-sectional study conducted among 754 Japanese high school students, adolescents with higher levels of physical activity reported fewer premenstrual symptoms [74].

Nutritional factors and eating behaviors have increasingly become a focus of PMS research. Among adolescents, PMS has been associated with a higher prevalence of disordered eating, including emotional and uncontrolled eating, while PMS and PMDD have also been associated with a greater prevalence of eating disorder symptoms. However, current evidence does not establish a causal relationship [96].

Moreover, several studies have suggested associations between PMS and selected micronutrients, particularly calcium, magnesium, vitamin D, and B vitamins, although the available evidence remains limited [95]. Evidence regarding calcium intake in adolescents with PMS remains limited. In a study by Derman et al., adolescent girls with PMS reported lower consumption of milk, yogurt, and cheese than girls without PMS, while no participants reported additional calcium supplementation. Higher milk consumption was significantly associated with lower severity of abdominal bloating, cramps, food cravings, and increased appetite. The authors suggested that further studies of calcium supplementation in adolescents with PMS are warranted [54].

Nevertheless, the available evidence remains insufficient to support specific dietary recommendations or routine supplementation strategies for the management of PMDs. Further well-designed prospective studies are required to clarify the role of nutrition and nutritional interventions in the prevention and treatment of PMDs.

### 7.4. Physiotherapy

Growing recognition of the multidimensional nature of PMDs has stimulated interest in complementary and supportive interventions that extend beyond pharmacological treatment. These approaches aim to address not only physical symptoms but also the psychological, behavioral, and functional consequences of premenstrual disorders, reflecting the increasingly accepted biopsychosocial model of management of PMDs [94].

Among supportive interventions, physiotherapy has emerged as a potentially valuable adjunctive treatment for PMS. A recent systematic review and meta-analysis of studies published between 2013 and 2023 reported that physiotherapy interventions significantly reduced depressive symptoms and were associated with improvements in pain and quality of life. The included studies assessed outcomes using the Visual Analog Scale, Premenstrual Syndrome Scale, Numeric Pain Rating Scale, and Beck Depression Inventory [97]. Although the available evidence remains limited by methodological heterogeneity and variation in intervention protocols, these findings suggest that physiotherapy may contribute to symptom reduction as part of a broader multidisciplinary treatment strategy.

**Figure 2:**
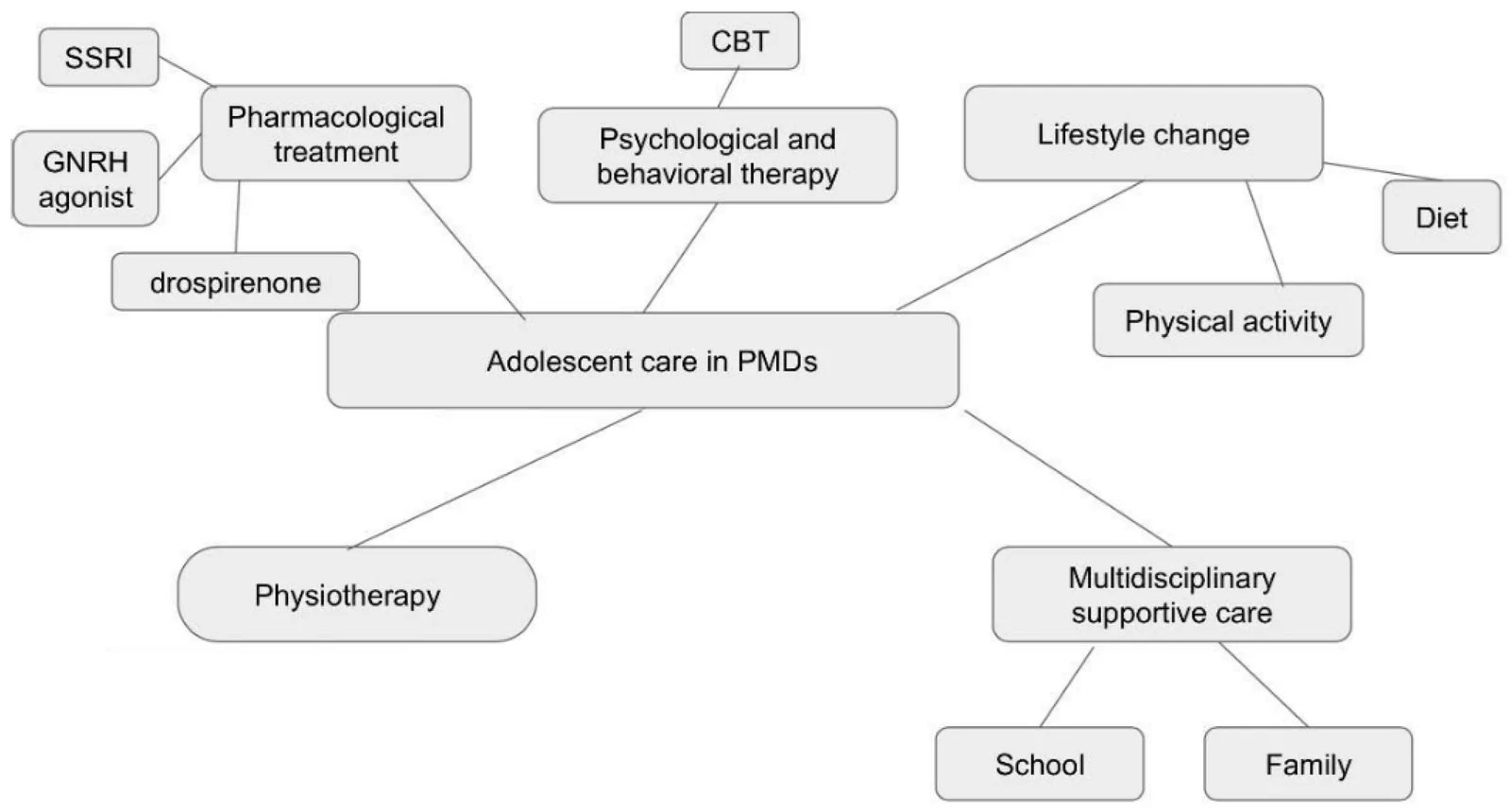
Multimodal management approaches for PMDs in adolescents.

In adolescents, direct evidence is more limited but encouraging. In a randomized study of adolescents with PMS, both resistive exercise and whole-body vibration performed three times weekly for 12 weeks were associated with significant reductions in anxiety, depressive symptoms, hyperhydration symptoms, cramps, and other premenstrual symptoms. No significant differences were observed between the two exercise interventions, suggesting that both active and passive forms of muscular training may have comparable effects on PMS symptom burden in adolescents. However, interpretation is limited by the use of self-reported symptom assessment and the relatively short study duration, and further research is needed to establish the long-term effectiveness of these interventions [98] (Figure 2).

## 8. Limitations

Several limitations should also be acknowledged. The available evidence on PMDs in adolescents remains limited in several areas. Most knowledge regarding the neuroendocrine mechanisms of PMDs is based on studies conducted in adult women. Similarly, evidence regarding sexual health and psychosexual functioning is scarce, with most available studies conducted in adult women. Evidence supporting therapeutic interventions is also predominantly derived from adult populations, and it remains unclear whether interventions demonstrated to be effective in adults have similar efficacy, feasibility, and acceptability during adolescence. These limitations indicate that caution is warranted when extrapolating findings from adult women to adolescents. Future research should therefore prioritize adequately powered adolescent trials using standardized diagnostic criteria, prospective symptom monitoring, and functional outcomes.

Interpretation of the available data is complicated by considerable heterogeneity in the diagnosis of PMS and PMDD, symptom assessment instruments, and study populations, limiting direct comparisons across studies and contributing to variability in reported prevalence and clinical outcomes. 

The narrative design of this review also has methodological limitations. No formal risk-of-bias assessment was performed, and both original studies and review articles were included to provide a comprehensive overview of the topic, which may have resulted in overlap between primary evidence and secondary interpretations. Finally, the literature search was restricted to publications in English and Polish, potentially excluding relevant studies published in other languages.

## 9. Conclusions

PMDs in adolescence should be understood as multidimensional conditions emerging at the intersection of biological, psychological, developmental, and sociocultural factors. Their impact extends beyond cyclical physical and emotional symptoms to affect mental health, interpersonal relationships, school functioning, and quality of life. The developmental context of adolescence, including maturation of the reproductive axis as well as the overlap between premenstrual and psychiatric symptoms, further complicates recognition and diagnosis.

An interdisciplinary approach is therefore essential for effective identification and management of PMDs, integrating gynecological, psychiatric, psychological, endocrinological, primary care, and lifestyle perspectives. Prospective assessment of symptom cyclicity and functional impairment remains central to diagnosis, while treatment should be individualized according to symptom severity, comorbidities, developmental needs, and patient preferences. Importantly, the current evidence base remains predominantly derived from adult populations. Future adolescent-focused research should clarify the biological and psychosocial determinants of PMDs and establish evidence-based, developmentally appropriate approaches to their diagnosis and multidisciplinary management.

## Figures and Tables

**Table 1:** Summary of selected studies relevant to premenstrual disorders in adolescents.

Study	Population/Age Range	Method	Main Outcome	Principal Findings
The Premenstrual Symptoms Screening Tool revised for adolescents: prevalence of severe PMS and PMDD in adolescents [53]	Adolescent girls, aged 12–18 years	PSST-A; cross-sectional screening	Prevalence and severity of severe PMS and PMDD symptoms assessed with the PSST-A	A total of 8.3% of adolescents experienced symptoms suggestive of PMDD; 21.3% experienced severe PMS. Irritability/anger was the most commonly reported symptom.
PMS in adolescents: severity and impairment [73]	Adolescent girls, aged 13–18 years	Symptom questionnaire, symptom-timing assessment, functional impairment ratings, and brief medical history	Severity, timing, and functional impairment associated with adolescent PMS	A total of 31% met the study criteria for PMS. Mood swings, anxiety, and irritability were the most severe symptoms, and the greatest impairment occurred in the home/family domain.
PMS and associated symptoms in adolescent girls [54]	Adolescent girls, aged 10–17 years	Questionnaire assessing PMS criteria, dysmenorrhea, menstrual regularity, and nutrition	Frequency and severity of PMS-associated symptoms and their relationship with dietary factors	PMS was associated with dietary habits; higher milk intake was inversely related to several symptoms including bloating and cramps.
Prevalence and impact of PMS in adolescent schoolgirls in the United Arab Emirates [58]	Adolescent girls, aged 12–18 years	Cross-sectional survey using multistage stratified cluster sampling	Prevalence of PMS and its impact on quality of life, school performance, social interactions, lifestyle, and emotional well-being	PMS had a moderate but significant negative impact on quality of life (*p* < 0.001), particularly school performance, social interactions, lifestyle, and emotional well-being. The authors concluded that PMS was prevalent but undertreated in this population.
The relationship between sleep and menstrual problems in early adolescent girls [44]	Adolescent girls; mean age 13.03 years	Self-reported menstrual characteristics; self- and caregiver-reported sleep measures	Association between sleep behavior and menstrual problems in early adolescence	Poorer sleep quality, shorter sleep duration, greater daytime sleepiness, and later sleep timing were associated with more severe premenstrual symptoms, greater menstrual pain, and greater daily-life impact of menstrual problems.
Correlates of PMS in Polish adolescents—Results from the POLKA 18 Study [12]	Adolescent girls, aged 18–19 years	Cross-sectional questionnaire study	Prevalence of PMS and its associations with mental health, perceived stress, and related health outcomes	PMS was reported by more than one-third of participants. Adolescents with PMS reported higher stress levels and poorer mental health outcomes than those without PMS.
Health-related quality of life among adolescents with premenstrual disorders: a cross-sectional study [52]	Adolescent girls, aged 14–19 years	Health-related quality of life assessed with the SF-36	Health-related quality of life in adolescents with premenstrual disorders, particularly PMDD	Students with PMDD had significantly lower SF-36 scores in all domains except physical functioning.
PMS health-related quality of life and psychiatric comorbidity in a clinical adolescent sample [67]	Adolescent girls, aged 13–18 years	K-SADS-PL for psychiatric diagnoses; Premenstrual Assessment Form for PMS severity; PedsQL for health-related quality of life	Health-related quality of life and psychiatric comorbidity in adolescents with PMS	Adolescents with PMS had lower health-related quality of life, and poorer quality of life was associated with increasing symptom severity. Psychiatric comorbidity, particularly anxiety and major depressive disorder, was common.
Attitude towards menstruation, prevalence of PMS and coping mechanisms among adolescent girls in Kerala, India: a cross-sectional study [63]	Adolescent girls, aged 13–19 years	Menstrual Attitude Questionnaire and Premenstrual Symptoms Screening Tool	Menstrual attitudes, prevalence/severity of PMS, coping mechanisms, and factors associated with negative attitudes and severe PMS	A total of 51% of individuals reported a negative attitude toward menstruation. Severe PMS was reported by 36.8%. Severe PMS was associated with negative menstrual attitudes, nuclear family structure, Muslim religion, and lower maternal education. Muslim religion and nuclear family structure were also associated with negative menstrual attitudes.
PMS and dysmenorrhea: urban–rural and multiethnic differences in perception, impacts, and treatment seeking [65]	Adolescent girls, aged 13–19 years	Focus-group discussions exploring perceptions, impacts, knowledge, and treatment-seeking behavior	Perceptions of menstruation-related problems, their impact, menstrual knowledge, and treatment-seeking across urban/rural and ethnic groups	Many participants lacked detailed menstrual knowledge before menarche and described menarche as shocking. PMS and dysmenorrhea were often normalized, professional help-seeking was uncommon, and rural girls more often reported embarrassment discussing menstrual problems with mothers or physicians.
Associations between physical activity, premenstrual symptoms, and psychological well-being among Japanese high school students: a cross-sectional study [74]	Adolescent girls	Premenstrual Symptoms Questionnaire and measures of self-esteem, loneliness, and psychological distress; physical activity categorized as high vs. non-high	Associations of physical activity with premenstrual symptom severity and psychological well-being	The high-physical-activity group had significantly lower PSQ scores, lower loneliness, and higher self-esteem. Greater physical activity was also associated with less PMS-related disruption of academic performance, relationships, and social activities.
Does premenstrual syndrome (PMS) affect the lifestyle of adolescent/young college students? [75]	Female university students	Analytical cross-sectional study; multistage cluster sampling; PSST and Health-Promoting Lifestyle Profile	Association between PMS severity and health-promoting lifestyle	A total of 81.1% individuals experienced severe PMS symptoms. Lifestyle scores were significantly lower in the PMS group, and PSST scores were negatively correlated with lifestyle scores, indicating a substantial adverse association between PMS and lifestyle.
Oral contraceptives containing drospirenone for PMS [76]	Women of reproductive age	Systematic review of randomized trials comparing drospirenone-containing combined oral contraceptives with placebo or other combined oral contraceptives	Effectiveness and safety of drospirenone-containing combined oral contraceptives for premenstrual symptoms	Drospirenone plus ethinyl estradiol may improve overall premenstrual symptoms and functional impairment compared with placebo, including lower Daily Record of Severity of Problems scores.

## Data Availability

No new data were created or analyzed in this study. Data sharing is not applicable to this article.

## Abbreviations

The following abbreviations are used in this manuscript:

CBT	Cognitive-behavioral therapy
COCs	Combined oral contraceptives
DSM-5	Diagnostic and Statistical Manual of Mental Disorders, Fifth Edition
GnRH	Gonadotropin-releasing hormone
PMDD	Premenstrual dysphoric disorder
PMDs	Premenstrual disorders
PMS	Premenstrual syndrome
PSST	Premenstrual Symptoms Screening Tool
PSST-A	Premenstrual Symptoms Screening Tool-Modified for Adolescents
SSRIs	Selective serotonin reuptake inhibitors
